# Modulation of T-bet and Eomes during Maturation of Peripheral Blood NK Cells Does Not Depend on Licensing/Educating KIR

**DOI:** 10.3389/fimmu.2016.00299

**Published:** 2016-08-24

**Authors:** Amandine Pradier, Federico Simonetta, Sophie Waldvogel, Carine Bosshard, Jean-Marie Tiercy, Eddy Roosnek

**Affiliations:** ^1^Division of Hematology, Department of Medical Specialties, Geneva University Hospitals, University of Geneva, Geneva, Switzerland; ^2^Transplantation Immunology Unit, National Reference Laboratory for Histocompatibility, Department of Genetics and Laboratory Medicine, University Hospital Geneva, Geneva, Switzerland

**Keywords:** NK cell, maturation, KIR, licensing, T-Bet, Eomes

## Abstract

Peripheral natural killer (NK) cells upregulate T-bet and downregulate Eomes, the key transcription factors regulating NK cell maturation and function during the last maturation steps toward terminally differentiated effector cells. During this process, NK cells acquire killer immunoglobulin-like receptors (KIR) and effector functions, such as cytotoxicity and target cell-induced cytokine production. Inhibitory KIR are pivotal in the control of effector functions, but whether they also modulate T-bet/Eomes expression is unknown. We have measured T-bet/Eomes levels, KIR expression, and effector functions of maturing CD94^neg^CD56^dim^NK cells using CD57 as surface marker for maturation. Our cohort consisted of 23 healthy blood donors (HBD) homozygous for the KIR A haplotype that contains only inhibitory KIR2DL1 (ligand HLA-C2), KIR2DL3 (ligand HLA-C1), and KIR3DL1 (ligand HLA-Bw4). We confirm that during maturation of NK cells, the number of KIR increases, levels of T-bet/Eomes are modulated, and that cells acquire effector functions, such as cytotoxicity (CD107) and target cell-induced cytokine production (TNF-α). Because maturation was associated with the increase of the number of KIR as well as with the modulation of T-bet/Eomes, the number of KIR correlated with the extent of T-bet/Eomes modulation. However, whether the KIR were triggered by their cognate HLA ligands or not had no impact on T-bet and Eomes expression, indicating that modulation of T-box transcription factors during NK cell maturation does not depend on signals conveyed by KIR. We discuss the relevance of this finding in the context of models of NK cell maturation while cautioning that results obtained in a perhaps quite heterogeneous cohort of HBD are not necessarily conclusive.

## Introduction

Natural killer (NK) cells are involved in the early response to pathogens as well as in the recognition of autologous cells under stress induced by infection or transformation (1). Maturation toward an end-stage cytotoxic effector cell is associated with the loss of CD94 and acquisition of killer immunoglobulin-like receptors (KIR), of markers such as CD57 and of fine-tuning of effector functions (2–5). Activation of NK cells depends on an aggregate of signals conveyed by various activating and/or inhibitory receptors. Responsiveness to self is reduced through the triggering of inhibitory KIR by their ligands that consist of allelic variants of MHC molecules that are clustered in three groups, HLA class I ligands (HLA-C1, HLA-C2, and HLA-Bw4). NK cells without inhibitory KIR for self may remain harmless either because acquisition of the most potent effector functions (arming) requires interaction of inhibitory KIR with self-ligands or because NK cells stimulated by activating receptors in the absence of interaction of inhibitory KIR with self reduce responsiveness. These latter two processes are referred to as “licensing” (6, 7) and “education” (8, 9). The ensuing level of responsiveness may still be adapted to their immunological environment in mature NK cells, but the molecular mechanisms have not been established yet [recently reviewed in Ref. (10)].

T-bet and Eomesodermin (Eomes), two T-box transcription factors, are master regulators of T cell effector functions, including cytotoxicity and interferon-gamma (IFN-γ) production (11, 12). Furthermore, recent reports have shown that these two transcription factors also regulate maturation and function of NK cells. Murine (13) and human (14, 15) NK cells express T-bet and Eomes constitutively, and mice lacking both T-bet and Eomes are completely deprived of NK cells (16). Moreover, the two T-box transcription factors are modulated during NK cell differentiation (14, 17, 18) and are necessary for maintenance and differentiation of peripheral NK cells, while their deletion in mature NK cells results in reversion to an immature phenotype (16).

Mature NK cells express more licensing/educating KIR, are more cytotoxic, and proliferate less (2, 4). Whether they produce more cytokines after stimulation with target cells remains under debate (4, 19). Furthermore, they have downregulated Eomes and upregulated T-bet (14, 20). The concurrence of these processes has made it difficult to determine whether licensing is associated with, or the cause of the modulation of effector functions as well as T-bet/Eomes expression. It has been difficult to establish that signals conveyed by inhibitory KIR encountering their ligands induce maturation. Indeed, one could also argue that the increase in effector functions found to be correlated with the expression of such KIR simply reflects a licensing-independent concomitant NK cell maturation (4).

T-bet and Eomes induce NK cell maturation by suppressing CD27 and c-kit and upregulating S1P5 and KLRG1 [recently reviewed in Ref. (21)]. Hence, if signals through licensing receptors induce maturation, one would expect that the same signals would modulate the levels of T-bet and Eomes first. In this report, we measured T-bet and Eomes levels in maturing NK cells in 23 healthy blood donors (HBD) that were homozygous for the KIR A haplotype, which comprises only inhibitory KIR for their respective HLA-C or HLA-Bw4 ligands. We found that T-bet and Eomes were modulated in parallel with the increase of KIR, but whether the ligand for the KIR was present or not had no impact. This finding strengthens the model of a recently put forward (4), licensing-independent concomitant NK cell maturation.

## Materials and Methods

### DNA Extraction and KIR Genotyping

Killer immunoglobulin-like receptors genotyping was performed on DNA extracted from 400 μl of blood using QIAmp Blood Mini kit (Qiagen) with the KIR Genotyping SSP kit (Invitrogen) according to manufacturer’s instructions. The AA haplotype was defined as KIR2DL5A^neg^/KIR2DL5B^neg^/KIR2DS1^neg^/KIR2DS2^neg^/KIR2DS3^neg^/KIR2DS5^neg^/KIR2DS1^neg^.

### HLA Typing

HLA-A, B, C typing was performed by PCR-SSO hybridization on microbeads arrays (Luminex technology), using the LabType high definition reagents (One Lambda, Canoga Park, CA, USA). When required for the assignment of the HLA-C1 and -C2 groups, typing ambiguities were resolved by PCR-SSP using Olerup SSP kits (Milan Analytika AG, La Roche, Switzerland).

### FACS Analysis, Effector Function Tests

Peripheral blood mononuclear cells (PBMCs) from AA haplotype homozygous donors blood from the Geneva University Hospitals blood transfusion center who gave informed consent by signing a standard form approved by the hospital’s ethical commission were isolated from anticoagulated blood by Ficoll density gradient centrifugation. We performed FACS analysis with monoclonal antibodies specific for the following antigens: CD158a (FITC, HP-MA4, Biolegend), CD57 (PECF594, clone NK1, BD Biosciences), CD94 (PerCPCy5.5, clone HP-3D9, BD Biosciences), CD158b (PEVio770, clone DX27, Miltenyi), CD158e (Alexa700, clone DX9, Biolegend), CD3 (APCH7, clone SK7, BD Biosciences), CD56 (BV421, cloneHCD56, Biolegend), CD107a (BV605, clone H4A3, Biolegend), TNF-α (BV605, clone MAb11, Biolegend), IFN-γ (BV605, clone 4S.B3, Biolegend), Eomes (eFluor 660, clone WD1928, e-Bioscience), and T-bet (PE, clone 4B10, e-Bioscience).

Effector functions were measured by stimulating PBMC at 37°C with K562 (cytotoxicity, TNF-α production) or with IL2/12/18 (IFN-γ production) as described before (20). Anti-CD107a was added at the start of the culture set up to measure degranulation. After 1 h, GolgiStop and GolgiPlug (BD Biosciences) were added, and cells were cultured for a further 3 h (CD107 and TNF-α) or 5 h (IFN-γ detection).

Intracellular staining for TNF-α, IFN-γ, Eomes, and T-bet was performed overnight at 4°C on permeabilized cells with FoxP3/transcription factor staining buffer set (e-Bioscience). CD3^neg^CD56^neg^ lymphocytes in the same blood sample were considered as Eomes and T-bet negative cells.

**Figure 1:**
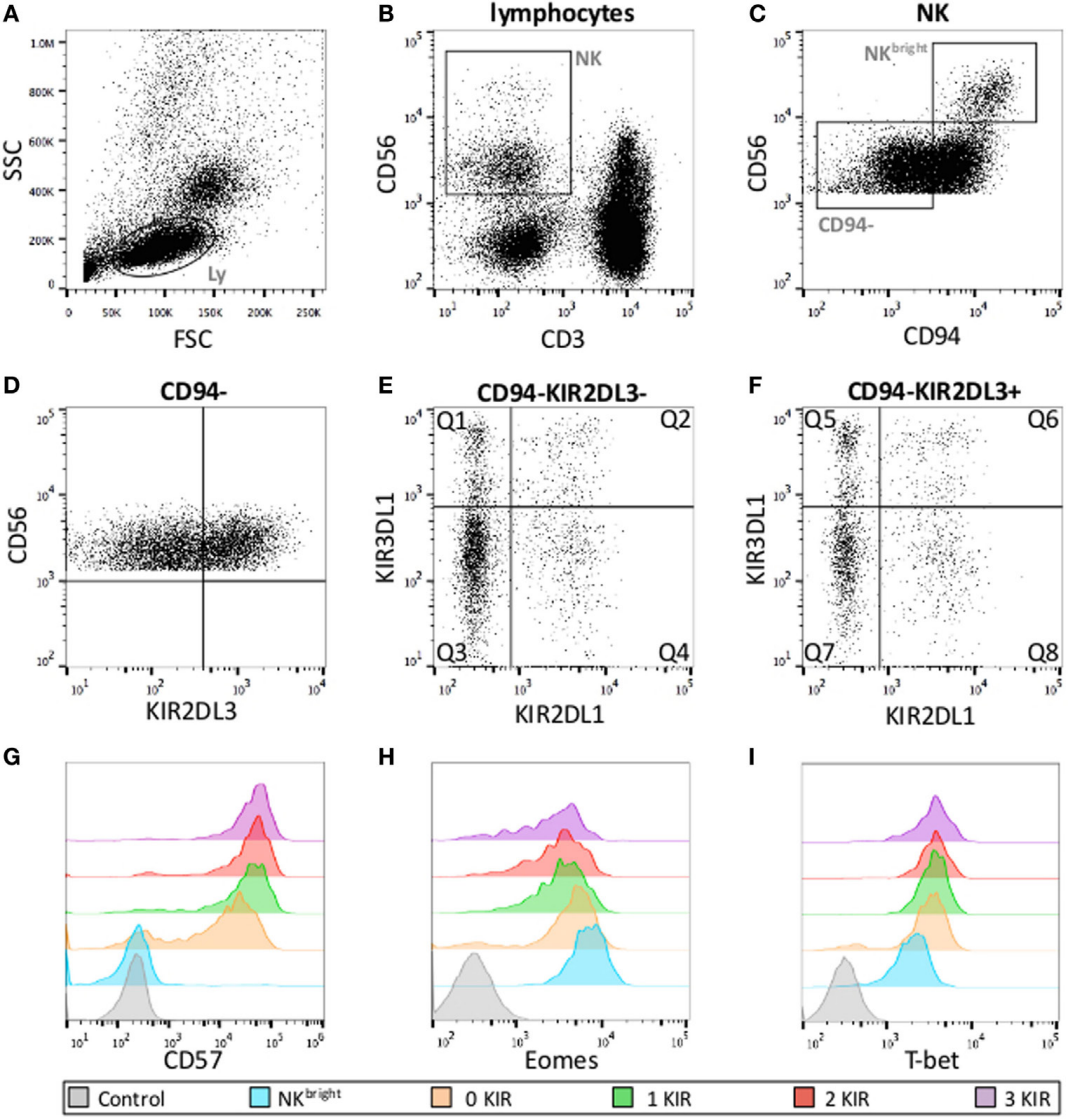
**Gating strategies for KIR subset analysis**. **(A)** Lymphocyte gates on FSC and SSC. **(B)** NK cell defined as CD56^pos^CD3^neg^ lymphocytes. **(C)** CD56^bright^ and CD56^dim^ NK cells according to the level of CD56-expression and gate on CD56^dim^CD94^neg^ NK cells used for further analysis. **(D–F)** CD56^dim^CD94^neg^ cells according to KIR expression (eight subsets). Q1: KIR2DL1^neg^KIR2DL3^neg^KIR3DL1^pos^, Q2: KIR2DL1^pos^KIR2DL3^neg^KIR3DL1^pos^, Q3: KIR2DL1^neg^KIR2DL3^neg^KIR3DL1^neg^, Q4: KIR2DL1^pos^KIR2DL3^neg^KIR3DL1^neg^, Q5: KIR2DL1^neg^KIR2DL3^pos^KIR3DL1^pos^, Q6: KIR2DL1^pos^KIR2DL3^pos^KIR3DL1^pos^, Q7: KIR2DL1^neg^KIR2DL3^pos^KIR3DL1^neg^, Q8: KIR2DL1^pos^KIR2DL3^pos^KIR3DL1^neg^. Examples of fluorescence intensity of CD57 **(G)**, Eomes **(H)** and T-bet **(I)** according to the number of KIR expressed with their respective negative controls.

Data acquired on a Gallios 3 cell analyzer (BD Biosciences) were analyzed with FlowJo software (Tree Star Inc.) with the gates depicted in Figure 1. The first two gates (Figures 1A,B) were used to demarcate NK cells as CD3^neg^CD56^pos^ cells with the FSC/SSC of lymphocytes. CD56^bright^ and CD56^dim^ NK cells were discriminated on basis of the level of CD56-expression (Figure 1C). Maturation state (CD57), effector functions, and T-bet/Eomes levels of CD94^neg^ (Figure 1D), CD56^dim^ NK cells were measured in the respective eight subsets defined by the expression KIR2DL1, KIR2DL3, and KIR3DL1 (Figures 1E,F). Levels of T-bet and Eomes are expressed as mean fluorescence intensity ratio (MFIR), representing the mean fluorescence of T-bet/Eomes divided by the fluorescence of T-bet/Eomes negative CD3^neg^CD56^neg^ cells in the same sample. The percentage of CD107^pos^ cells is expressed as %CD107^pos^ NK cell after stimulation with K562 – % CD107^pos^ unstimulated NK cell.

### Statistical Analysis

Non-parametric Wilcoxon matched-pairs signed rank test or Mann–Whitney test were used to compare groups, *p* values >0.05 were considered as not statistically significant.

## Results

We have studied maturing NK cells in 23 KIR A haplotype homozygous HBD that comprise only the inhibitory variant of KIR2DL1, KIR2DL3, and KIR3DL1. This allowed us to measure the effect of the presence or absence of HLA ligands on inhibitory KIR using monoclonal antibodies that also recognize their activatory variants. Table 1 shows the KIR and HLA class I molecules expressed by the 23 HBD tested. As expected for KIR A haplotype homozygous individuals, NK cell subpopulations expressing KIR2DL1 and KIR2DL3 were present in all HBD. Two of 23 HBD lacked NK cells expressing KIR3DL1, which is owed to the fact that some KIR3DL1 allelic variants are not expressed at the protein level (22). The last three columns of the table show in which HBD the respective KIR encounter their cognate ligands [KIR2DL1↔C2, KIR2DL3↔C1, and KIR3DL1↔Bw4 epitope on HLA-B antigens as well as the HLA-A antigens (23, 24) marked in bold]. Hence, KIR2DL1 encountered its cognate HLA ligand in 10/23 and KIR2DL3 in 22/23 HBD, while KIR3DL1 was able to license NK cells in 15/23 HBD.

**Table 1:** **HLA class I and KIR expression in the 23 HBD**.

HBD	HLA-A	HLA-A	HLA-B	HLA-B	Epitopes B	HLA-C	HLA-C	Epitopes C	KIR3DL1	KIR2DL3	KIR2DL1
1	02:01	**32:01**^c^	15:01	40:02	Bw6/Bw6	02:02	03:03	C1/C2	^a^	^a^	^a^
2	02:01	30:02	15:01	35:01	Bw6/Bw6	01:02	04:01	C1/C2		^a^	^a^
3	24:02	30:01	13:02	44:03	Bw4/Bw4	06:02	16:01	C1/C2	^a^	^a^	^a^
4	02:01	02:01	18:01	51:01	Bw4/Bw6	07:01	14:02	C1/C2	^a^	^a^	
5	02:01	11:01	18:03	39:09	Bw6/Bw6	07:01	07:02	C1/C2		^a^	
6	02:01	25:01	13:02	35:01	Bw4/Bw6	07:01	07:02	C1/C2	^a^	^a^	
7	01:02	02:01	07:02	49:01	Bw4/Bw6	07:01	07:02	C1/C2	^a^	^a^	
8	02:01	23:01	49:01	51:01	Bw4/Bw4	02:02	07:01	C1/C2	^a^	^a^	^a^
9	01:01	01:01	57:01	57:01	Bw4/Bw4	06:02	07:01	C1/C2	^a^	^a^	^a^
10	01:01	11:01	49:01	52:01	Bw4/Bw4	07:01	12:02	C1/C2	^a^	^a^	
11	02:01	32:01	35:01	44:02	Bw4/Bw6	04:01	05:01	C2/C2	^a^		^a^
12	01:01	11:01	15:01	35:01	Bw6/Bw6	03:03	04:01	C1/C2		^a^	^a^
13	02:01	03:01	56:01	56:01	Bw6/Bw6	01:02	01:02	C1/C2		^a^	
14	02:01	24:02	15:01	44:02	Bw4/Bw6	03:03	05:01	C1/C2	^a^	^a^	^a^
15	03:01	29:02	07:02	44:03	Bw4/Bw6	07:02	16:01	C1/C2	^a^	^a^	
16	02:01	26:01	13:02	58:01	Bw4/Bw4	06:02	07:01	C1/C2	^b^	^a^	^a^
17	24:02	30:02	38:01	40:01	Bw4/Bw6	03:04	12:03	C1/C2	^b^	^a^	
18	01:01	11:01	08:01	51:01	Bw4/Bw6	07:01	15:02	C1/C2	^a^	^a^	^a^
19	26:01	32:01	38:01	44:03	Bw4/Bw4	12:03	16:01	C1/C2	^a^	^a^	
20	02:01	24:02	35:01	44:02	Bw4/Bw6	01:02	01:02	C1/C2	^a^	^a^	
21	26:01	33:01	14:02	44:02	Bw4/Bw6	08:02	12:03	C1/C2	^a^	^a^	
22	01:01	68:01	08:01	39:01	Bw6/Bw6	07:01	12:03	C1/C2		^a^	
23	23:01	31:01	07:02	08:01	Bw6/Bw6	07:01	07:02	C1/C2		^a^	

*^a^Licensed*.

*^b^KIR3DL1 not expressed (22)*.

^c^HLA-A*3201 with Bw4 epitope (23, 24).

### Expression of Eomes/T-bet/CD57 in Relation to the Number of KIR

**Figure 2:**
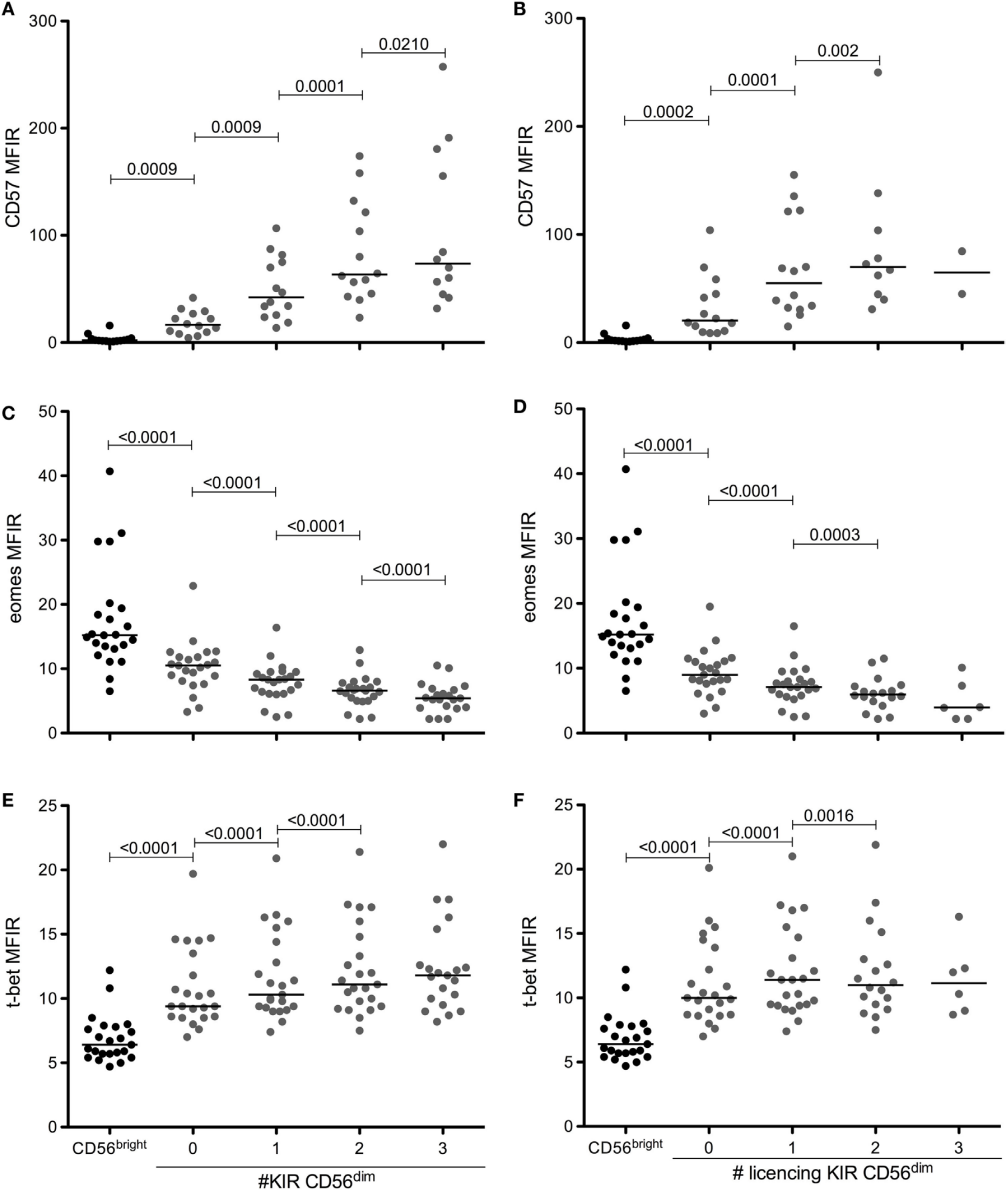
**CD57 and T-bet are upregulated, while Eomes is downregulated during NK cell maturation and KIR acquisition**. Expression levels of CD57 **(A,B)**, eomes **(C,D)**, and T-bet **(E,F)** in CD56^bright^ or in CD56^dim^ CD94^neg^ cells according to the number of KIR **(A,C,E)** or licensing KIR **(B,D,F)** expressed. Medians are shown, and Wilcoxon matched-pairs signed rank test was used for statistical analysis, *p* values are indicated. As mentioned in the text, CD94^pos^ NK cells have been excluded from analysis because, although the results are very similar (not shown), the inclusion of CD94^pos^ NK cells would render the interpretation of the results more difficult.

Several reports have shown that NK cells upregulate CD57 and increase the number of KIR expressed during maturation. In parallel, CD56^dim^ NK cells downregulate Eomes and upregulate T-bet. Using the gating strategy shown in Figure 1, we measured the levels of CD57, Eomes, and T-bet in CD56^bright^ NK cells and in the eight NK cell subpopulations of CD56^dim^CD94^neg^ NK cells expressing different combinations of KIR2DL1, KIR2DL3, and KIR3DL1. Figure 2 shows the results of our panel of 23 HBD and confirms (Figures 2A,B) that indeed CD57 levels on CD94^neg^KIR^pos^CD56^dim^ NK cells are significantly higher than on CD94^neg^KIR^neg^CD56^dim^ NK cells, and that CD57 levels increase further with the number of KIR expressed (for comparison, CD57, Eomes and T-bet levels on CD56^bright^ NK cells are shown). This was true when all KIR (Figure 2A) or only licensing KIR (Figure 2B) were considered. Furthermore, levels of Eomes that are significantly lower in CD56^dim^ NK cells than in CD56^bright^ NK cells (Figures 2C,D) are further downregulated with the acquisition of KIR. As expected, T-bet levels were the opposite of Eomes levels. CD56^dim^ NK cells expressed more T-bet than their CD56^bright^ counterparts, and these levels increased with the number of KIR expressed (Figures 2E,F). Again, whether all KIR were licensing (Figures 2D,F) or not (Figures 2C,E) did not seem to have a considerable impact.

### Eomes/T-bet/CD57 Expression and Effector Function of Licensed and Unlicensed NK Cells

**Figure 3:**
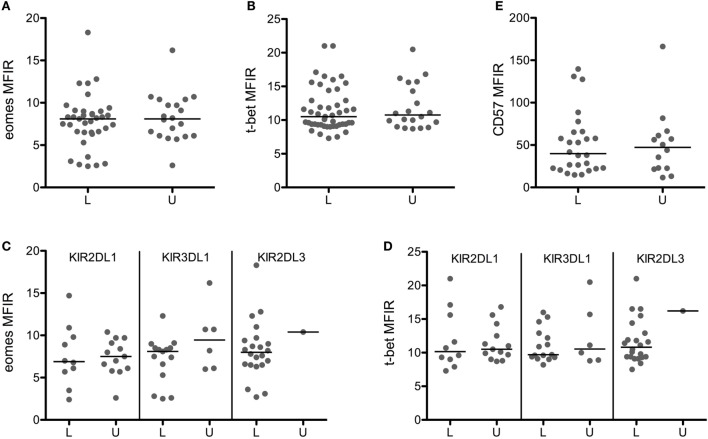
**Licensing has no effect on eomes, T-bet, and CD57 expression**. Eomes **(A)**, T-bet **(B)**, and CD57 **(E)** MFIR are shown in CD56^dim^CD94^neg^ cells expressing a single licensed (L) or unlicensed (U) KIR. Eomes **(C)** and T-bet **(D)** MFIR are represented for each KIR separately. Medians are shown, and Mann–Whitney test was used for statistical analysis.

To investigate the effect of licensing on Eomes/T-bet levels more precisely, we gated on CD56^dim^ NK cells expressing a single KIR and tested the impact of the presence or absence of its HLA ligand. Furthermore, we gated on CD94^neg^ NK cells to disregard the weaker inhibitory signals through CD94/NKG2A (25), of which the contribution to licensing is unclear. Figures 3A,B show that the expression of Eomes/T-bet in licensed NK cells was identical to the level in unlicensed NK cells. This was true for single KIR2DL1^pos^ NK cells in the presence (HBD 1–3, 8, 9, 11, 12, 14, 16, 18) or in the absence (HBD 4–6, 7, 10, 13, 15, 17, 19–23) of its ligand HLA-C2 (Figure 3C) as well as for single KIR3DL1^pos^ NK cells in the presence (HBD 1, 3, 4, 6–11, 14, 15, 18–21) or in the absence (HBD 2, 5, 12, 13, 16, 17, 22, 23) of its ligand HLA-Bw4 (Figure 3D). We were not able to compare the effect of the ligand HLA-C1 on single KIR2DL3^pos^ NK cells because our panel comprised only one HLA-C1 negative HBD (HBD 11), but the fact that the Eomes/T-bet levels in licensed single KIR2DL3^pos^ NK cells (HBD 1–10, 12–23) was identical to the levels in (un)licensed single KIR2DL1^pos^ or KIR3DL1^pos^ NK cells did suggest that the effect of licensing in single KIR2DL3^pos^ NK cells may also be negligible.

**Figure 4:**
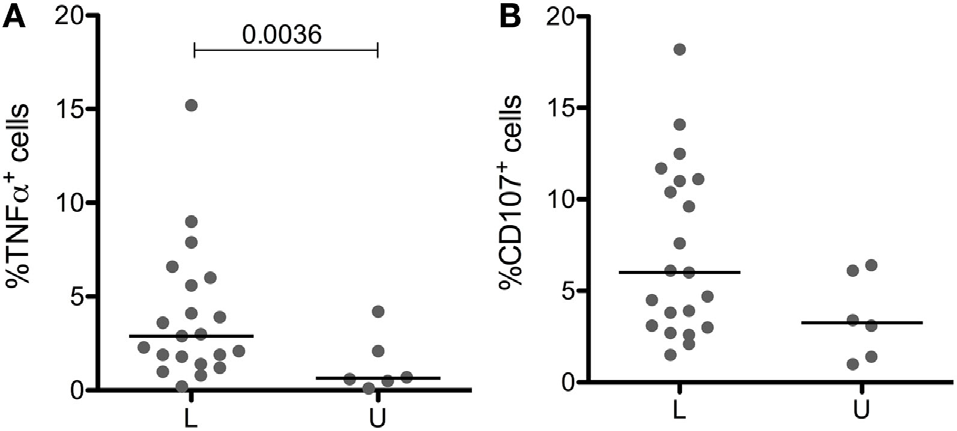
**Impact of licensing on TNF-α production and cytoxicity in CD56^dim^CD94^neg^ cells**. TNF-α^pos^ **(A)** and CD107^pos^ **(B)** cells in CD56^dim^CD94^neg^ cells expressing a single licensed (L) or unlicensed (U) KIR after culture for 3 h in presence of K562 target cells. Medians are shown, and Mann–Whitney test was used for statistical analysis, *p* values are indicated when significant.

Licensed and unlicensed NK cells expressed similar levels of CD57 (Figure 3E), but effector functions that were higher in cells expressing more KIR (data not shown) were to some extent affected by licensing. Licensed NK cells produced significantly more TNF-α than unlicensed NK cells (Figure 4A), and the most cytotoxic NK cells (Figure 4B) were licensed. Hence, we found that the effect of licensing on maturation and effector functions of human NK cells may be of secondary importance only, which in our opinion accords well with the variegated results on the effect of licensing on different effector functions of human NK cells reported by others (2, 4, 19).

## Discussion

The expression of the master regulators of NK cell differentiation T-bet and Eomes is modulated during the maturation toward end-stage cytotoxic NK cells. Cytokine-producing CD56^bright^ NK cells express higher levels of Eomes and lower levels of T-bet than cytotoxic CD56^dim^ NK cells, and the T-bet/Eomes ratio increases further during the last maturation steps toward terminally differentiated CD56^dim^CD57^pos^ NK cells (14, 15, 20). During maturation, the number of KIR increases, markers such as CD94 and CD62L are downregulated, CD57 is upregulated, and effector functions change (2–5).

There are indications that licensing favors maturation, but the extent thereof remains under debate (2, 4, 19). It is conceivable that licensing modulates T-bet and Eomes and that the ensuing upregulation of T-bet is at the origin of the increased cytotoxicity that is characteristic of mature NK cells. However, it may also be that licensing, upregulation of T-bet, and the increase of cytotoxicity are maturation-associated, parallel processes.

To answer this question, we measured T-bet and Eomes levels in maturing NK cells that were licensed or not. We found no differences in T-bet/Eomes levels in licensed or unlicensed NK cells. Although this would indicate that licensing and modulation of T-bet and Eomes are independent processes, we remain somewhat reluctant to draw such a firm conclusion based on the analysis of a panel of perhaps quite heterogeneous HBD. In humans, it might not be that easy to discriminate between cells that have matured in response to signals through licensing KIR or in response to cytokines (6, 7, 26, 27) produced during an inflammatory immune response. Indeed, mouse models have been somewhat more consistent regarding the effect of licensing on effector functions (6, 28, 29) than human studies (2, 4, 8, 19, 30). Likewise, our results regarding the acquisition of effector functions or of a more mature phenotype of CD56^dim^CD94^neg^ single KIR^pos^ NK cells in the presence or absence of their cognate HLA ligand were not very conclusive. Licensed NK cells produced more TNF-α, seemed to be somewhat more cytotoxic but did not express more CD57 than unlicensed cells. We also measured differences in IFN-γ production, the cytokine that is commonly tested in functional studies of NK cells. We found no differences, but again, we consider these results with caution because in our hands only few NK cells *ex vivo* produce IFN-γ when stimulated with target cells without being cultured in the presence of IL2/IL-12/IL-18 (20). Licensed and unlicensed CD56^dim^ NK cells produced similar quantities of IFN-γ after stimulation with cytokines (data not shown), but this result is not more than had to be expected because the culture with cytokines would simply annul potential differences between licensed and unlicensed NK cells.

## Conclusion

In conclusion, we found no impact of licensing on the expression of T-bet and Eomes.

Because T-bet and Eomes are the master regulators of NK cell maturation, these results are hard to reconcile with the model in which licensing induces maturation directly. However, because we also found that the effect of licensing was not easily substantiated for classical maturation-associated attributes, such as cytokine production, cytotoxicity, and expression of CD57, we believe that this finding should be interpreted with caution. In fact, human NK cells may not be ideal to study the effect of licensing on maturation because the circumstances *in vivo* that may bypass the need for licensing are hard to define.

## Author Contributions

AP, FS, and ER designed the study; AP and CB performed the experiments; SW organized the cohort of HBD; J-MT helped with HLA and KIR-typing; AP and ER wrote the manuscript.

## Conflict of Interest Statement

The authors declare that the research was conducted in the absence of any commercial or financial relationships that could be construed as a potential conflict of interest.
